# Prognostic significance of right ventricular dysfunction in patients presenting with acute left-sided heart failure

**DOI:** 10.1186/s43044-023-00432-8

**Published:** 2024-01-02

**Authors:** Mirna M. Shaker, Hesham S. Taha, Hossam I. Kandil, Heba M. Kamal, Hossam A. Mahrous, Ahmed A. Elamragy

**Affiliations:** Department of Cardiology, Faculty of Medicine, Cairo University, 27 Nafezet Sheem El Shafae St Kasr Al Ainy, Cairo, 11562 Egypt

**Keywords:** Acute heart failure, Right ventricular dysfunction, Echocardiography, Cardiovascular death, Heart failure hospitalization

## Abstract

**Background:**

The prognostic value of right ventricular (RV) function in chronic heart failure (HF) has lately been well established. However, research on its role in acute heart failure (AHF) is sparse.

**Results:**

This study comprised 195 patients, aged between 18 and 80 years, with acute left-sided heart failure (HF) and a left ventricular ejection fraction (LVEF) < 50%. Patients with LVEF ≥ 50%, mechanical ventilatory or circulatory support, poor echocardiographic windows, prosthetic valves, congenital heart diseases, infective endocarditis, and/or life expectancy < 1 year due to non-cardiac causes were excluded. The study participants’ mean age was 57.7 ± 10.9 years, and 74.9% were males. Coronary artery disease was present in 80.5% of patients. The mean LVEF was 31% ± 8.7. RV dysfunction (RVD), defined as tricuspid annular plane systolic excursion (TAPSE) < 17 mm, RV S' < 9.5 cm/s and/or RV fractional area change (FAC) < 35%, was identified in 48.7% of patients. The RV was dilated in 67.7% of the patients. RVD was significantly associated with a longer HF duration, atrial fibrillation, and idiopathic dilated cardiomyopathy. The primary outcome, a 6-month composite of cardiovascular death or hospitalization for worsening HF (HHF), occurred in 42% of the participants. Cardiovascular mortality and HHF occurred in 30.5% and 23.9% of the patients, respectively. The primary endpoint and longer CCU stays were significantly more common in patients with RVD than in those with normal RV function. RV dilatation was significantly associated with the primary outcome, whether alone or in combination with RVD. Multivariate regression analysis showed that only RV global longitudinal strain (GLS) independently predicted poor outcomes.

**Conclusions:**

RVD and RV dilatation strongly predict CV death and HHF in patients with AHF and LVEF < 50%. Multivariate analysis showed that RV GLS was the only predictor of a composite of CV death and HHF.

## Background

Despite significant advances in heart failure (HF) management, acute HF (AHF) remains a devastating condition and a cause of frequent hospital admissions [1]. Right ventricular (RV) function is generally accepted as an essential prognostic factor in chronic HF. Nevertheless, few recommendations have emerged based on RV assessment, [2] which may be due to conflicting data on determinants of RV function, a limited understanding of the mechanisms leading to its impairment, and relatively limited evidence on its relation to outcomes [3].

Bedside focused heart ultrasound is the first-line modality for RV assessment in critically ill patients. In contrast, invasive hemodynamic assessment is indicated in case of resistance to treatment or inconclusive non-invasive tests [4]. The global RV function is usually assessed by quantitative evaluation of one or more of the following parameters: fractional area change (FAC), tricuspid annular plane systolic excursion (TAPSE), tissue Doppler imaging (TDI)-derived systolic S' velocity or RV myocardial performance index [5]. 

Speckle tracking imaging is a relatively new technology for assessing myocardial deformation and offers many advantages over conventional echocardiographic methods. Unlike pulsed wave and TDI, it is less dependent on angle or load, rapid, and more precise. Thus, it is a valid method for assessing RV mechanical changes, with results comparable to cardiac magnetic resonance (CMR) [5, 6].

In view of the peculiar shape of the RV, three-dimensional echocardiography (3DE) may have an advantage over two-dimensional echocardiography (2DE) in RV assessment [7].

CMR has become the gold standard for evaluating the ventricular function with high accuracy and reproducibility [8]. However, CMR use may be limited in vitally unstable patients or those with some cardiac implantable electronic devices [9, 10]. Therefore, echocardiography remains a necessary non-invasive tool to assess the cardiac function in those patients [11].

Given these facts, echocardiography is the first choice for assessing the RV in different cardiovascular diseases. Nevertheless, further research is required to highlight the diagnostic and predictive role of the RV evaluation by echocardiography in patients with AHF, particularly in our population.

In this registry, we studied the effect of right ventricular structure and function on acute left-sided heart failure clinical outcomes.

## Methods

### Study population

This was a prospective cohort study of all consecutive patients presenting with left-sided AHF and left ventricular ejection fraction (LVEF) < 50% in the cardiovascular department of our hospital between September 2019 and September 2021. Left-sided AHF was defined as the rapid onset or worsening of symptoms and/or signs of HF (orthopnea, paroxysmal nocturnal dyspnea, bilateral pulmonary rales, or manifestations of hypoperfusion) [12, 13]. Patients were defined as having heart failure with reduced ejection fraction (HFrEF) if LVEF was < 40% and HF with mildly reduced ejection fraction (HFmrEF) if LVEF was 40–49% [12]. Exclusion criteria included any of the following: LVEF ≥ 50%, age < 18 years or > 80 years, mechanical circulatory support, mechanical ventilation, inadequate image quality to assess RV parameters, life expectancy < 1 year due to non-cardiac factors such as advanced cancer, prosthetic valves, congenital heart disease, or infective endocarditis.

## Clinical assessment

We recorded patients’ clinical, electrocardiographic and laboratory data during hospital admission. Clinical data included age, gender, symptoms and signs of HF, duration of illness, possible underlying etiology, New York Heart Association (NYHA) functional class, height, weight, body mass index, heart rate and blood pressure (BP), history of hypertension (HTN), diabetes mellitus (DM), malignancy, coronary artery disease (CAD), cerebrovascular accidents (CVA), previous cardiovascular interventions, medications, and smoking status. Laboratory data included serum creatinine, serum sodium and potassium, cardiac enzymes, and a complete blood count. The N-terminal pro-brain natriuretic peptide (NT-proBNP) level was determined according to the local standard of care. Definitions of the clinical characteristics were: HTN –defined as either a systolic and/or diastolic elevation of BP (˃140/90 mm Hg) or current antihypertensive pharmacologic therapy [14], DM –defined as hyperglycemia, Hemoglobin A1C ≥ 6.5% or on current antidiabetic pharmacologic treatment [15], and smoking status –non-smoker, current smoker (active smoking within the past 12 months), or ex-smoker (quitting smoking for more than 12 months).

## Echocardiography

Transthoracic echocardiography was performed in all patients using a Philips EPIQ 7 unit (Phillips Medical Systems, Andover, MA, USA) equipped with a 1–5 MHz X5-1 transducer. Patients were examined in the left lateral decubitus position. Multiple short- and long-axis standardized echocardiographic views were taken according to the current American Society of Echocardiography guidelines to evaluate all segments of the RV: parasternal long axis, parasternal short axis, RV inflow, apical four-chamber, focused apical 4-chamber, and subcostal four-chamber views [5].

## Study of the right ventricle

### Assessment of RV structure [5, 16, 17]

A focused apical 4-chamber image was used to estimate the RV dimensions at end-diastole. The basal RV linear dimension was measured in its lower one-third. The mid-cavity RV transverse dimension was measured in the middle third at the same level as the papillary muscles. The RV length was also measured. The proximal right ventricular outflow tract (RVOT) was measured from the anterior RV wall to the interventricular septal-aortic junction in the parasternal long-axis view, and to the aortic valve in the parasternal short-axis view. Next, the diameter of the distal RVOT was measured proximal to the pulmonary valve in the parasternal short-axis view. A linear measurement of the RV wall thickness (either by M-mode or 2DE) was done at end-diastole, beneath the tricuspid annulus, at a distance approaching the length of the anterior tricuspid leaflet, when it was completely opened and parallel to the RV free wall. The trabeculae, papillary muscles, and epicardial fat were excluded. Zoomed-in imaging, focusing on the RV mid-wall and respiratory maneuvers improved endocardial delineation.

## Assessment of RV function [5, 16–19] (Fig. 1)

**Fig. 1:**
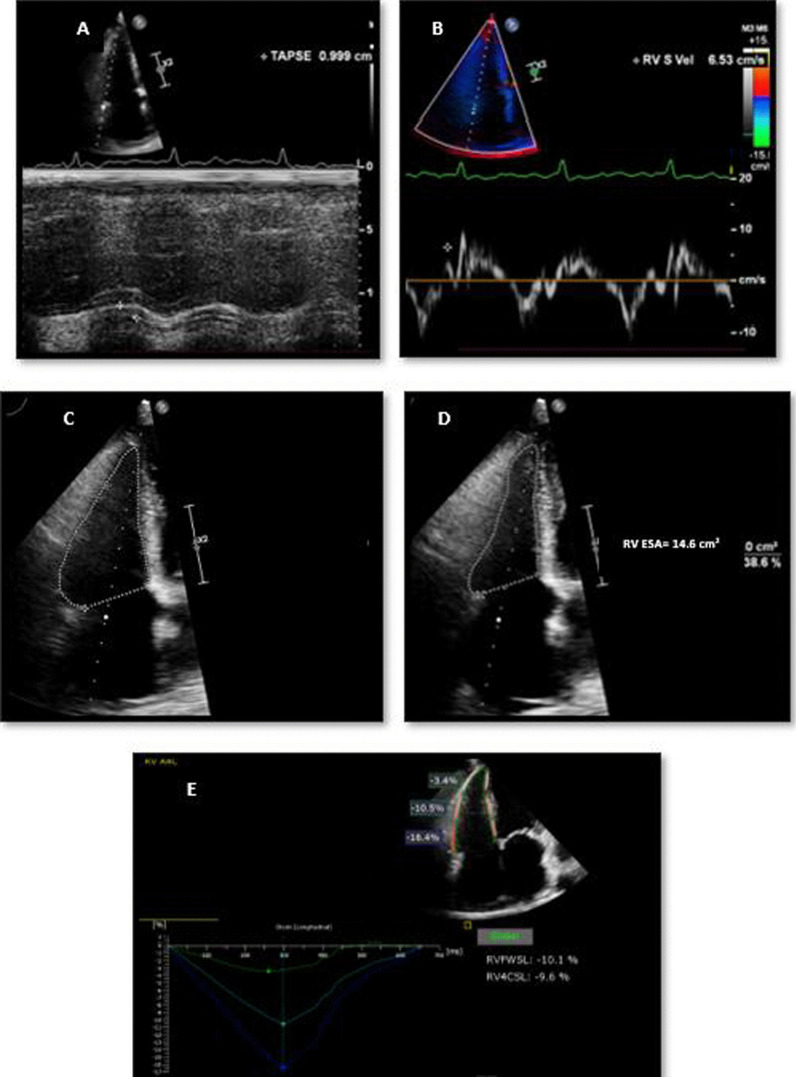
Methods of measurements of right ventricular (RV) systolic function. RV function was impaired by **A** tricuspid annular plane systolic excursion (TAPSE≈ 1 cm), **B** peak tricuspid annular systolic velocity by Pulsed-wave Tissue Doppler Imaging (S'≈ 6.5 cm) **C** & **D** RV fractional area change (FAC = 32%) calculated from RV end-diastolic area (RV EDA) and RV end-systolic area (RV ESA) and by **E** longitudinal RV free wall longitudinal strain (RV FWS = − 10.1%) and right ventricular global longitudinal strain (RV GLS = − 9.6%)

### Conventional 2D echocardiographic methods

For better visualization of the RV, a focused apical 4-chamber view was used to assess the RV function. TAPSE was measured using M-mode tracing obtained with the M-mode cursor aligned through the lateral side of the tricuspid annulus. Pulsed TDI on the lateral side of the tricuspid annulus was performed to measure the peak tricuspid annular S′ velocity. The RV myocardial performance (Tei) index was calculated as the ratio of isovolumetric relaxation time to isovolumetric contraction time divided by RV ejection time: [(isovolumetric relaxation time + isovolumetric contraction time) / ejection time]. Next, the RV endocardium was traced during systole and diastole to obtain the end-systolic and end-diastolic areas. We used the formula [100 × (RV end-diastolic area–RV end-systolic area/RV end-diastolic area)] to obtain RV fractional area change (FAC).

The right atrial (RA) minor axis was measured at end-systole as the distance between the lateral RA wall and interatrial septum at the mid-atrial level in the apical 4-chamber view. The RA blood-tissue interface was then traced, excluding the area under the tricuspid annulus, to measure the RA area.

In cases of atrial fibrillation (AF), the measurements were averaged over five cardiac cycles based on the guidelines.

### 2D-speckle tracking echocardiography

A 2D strain software was used to perform RV strain analysis. For offline analysis, B-mode pictures of the apical 4-chamber views were digitally recorded in a cine-loop manner. After manually tracing the RV endocardial border over one frame, the endocardial borders were traced automatically throughout the cardiac cycle. Myocardial velocity was calculated as the ratio of the speckle displacement from frame to frame and the time interval. RV global longitudinal strain (GLS) was obtained and averaged for all six RV free wall and septal segments. The longitudinal strain of the RV free wall (RV FWS) was measured from the three lateral wall segments (basal, mid, and apex), and that of the interventricular septum from the three septal segments. If the tracking was of poor quality, segments were discarded.

RVD was diagnosed if one or more of the following parameters were impaired: RV FAC, TAPSE and/or RV S' [5, 16, 17]. We did not rely on the Tei index as it could not be assessed in all patients due to the presence of AF [5, 16]. The patients were then classified into two groups according to the presence or absence of RVD.

### Measurements of other cardiac chambers

In all subjects, cardiac chamber quantification by 2DE was performed according to the guidelines [5]. Left ventricular (LV) diameters were measured using 2DE according to the recommended criteria. We measured the thicknesses of the interventricular septum and LV posterior wall at end-diastole. LV end-diastolic volume and end-systolic volume were obtained from apical 4- and apical 2- chamber imaging, and LVEF was calculated using the modified Simpson's method. Left atrial (LA) volume was also measured using the biplane method from the apical 4- and 2-chamber views. Grading of the severity of valvular stenosis or regurgitation was performed as per guidelines [20]. Mitral inflow velocities were determined by pulsed Doppler imaging. We assessed the peak early (E) and late (A) diastolic velocities, and the E/A ratio from the mitral inflow velocity pattern [20].

To prevent inter-observer variability, 5% of the studies were assessed by two experienced echocardiographers.

### Clinical endpoints

We followed the patients for six months by clinic visits and telephone calls. The primary endpoint was a composite of cardiovascular (CV) death or hospitalization for worsening HF (HHF) within six months. The secondary endpoints were the individual components of the primary endpoint, all-cause death, hospitalization for any cause, hospital length of stay (LOS), cardiac care unit (CCU) stay, occurrence of atherosclerotic CV complications including acute coronary syndrome (ACS), CVA, and vascular complications.

### Statistical analysis

Data were tested for normality. Continuous variables are presented as mean ± SD for normally distributed data and as median and interquartile range for skewed data. Categorical data are presented as numbers (%). Comparisons between the two groups were made using Chi-square and Fischer's exact tests for categorical data and the t-test or Wilcoxon-Sign test for normal and skewed data, respectively. All tests were two-sided, and *p* values were considered statistically significant if < 0.05, non-significant if ˃ 0.05, and highly significant if < 0.001.

We performed multivariate Cox regression analysis to identify the predictors of the occurrence of the primary endpoint.

### Ethical approval

All participants provided informed consent to participate in the study. This study was approved by the ethics committee of the University Hospital.

## Results

**Fig. 2:**
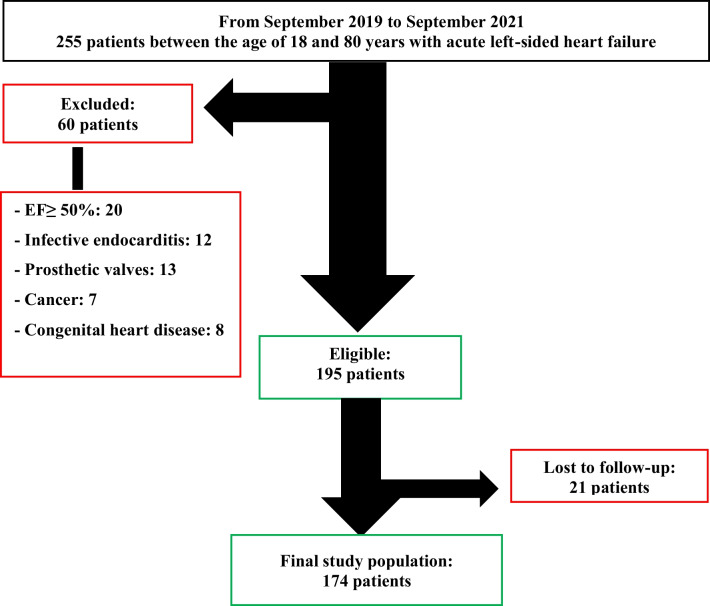
Patient selection flow chart

A total of 255 patients were screened for eligibility for the study, and 60 were excluded – LVEF ≥ 50%: 20, infective endocarditis: 12, prosthetic valves: 13, malignancy: 7, and congenital heart disease: 8. Only 195 patients met the inclusion criteria and were enrolled in this study. Of these, 21 were lost to follow-up. (Fig. 2).

## Baseline characteristics of the study population

The 195 eligible patients' initial baseline characteristics were evaluated. The mean age was 57.7 ± 10.9 years, and 74.9% were men. In 73.8% of the patients, the duration of the HF was less than one year, and in 6.7%, it was more than five years. HTN, DM, and CAD were prevalent in 49.7%, 54.4%, and 80.5% of the patients, respectively. There was a history of previous revascularization in 24.6% of the patients. (Table 1).

**Table 1:** Baseline clinical characteristics of the study population

Variables	Number (%) or mean ± SD (n = 195)
Age	57.7 ± 10.9
BMI (kg/m^2^)	27.5 ± 5.3
Male gender	146 (74.9)
*Smoking*	
Non-smoker	66 (33.8)
Current smoker	89 (45.6)
Ex-smoker	40 (20.5)
*HF duration*	
< 1 month	70 (43.8)
> 1 month—< 1 year	48 (30)
1–5 year(s)	31 (19.4)
> 5 years	11 (6.7)
*Comorbidities*	
HTN	97 (49.7)
DM	106 (54.4)
CAD	157 (80.5)
Previous PCI/CABG	48 (24.6)
Previous cardiac arrest	4 (2.1)
Chronic chest disease	46 (23.6)
Renal impairment	84 (43.1)
ESKD on regular dialysis	4 (2.1)
Previous CVA	25 (12.8)
Previous exposure to chemotherapy	4 (2.1)
Thyroid disorders	6 (3.1)
PAD	18 (9.2)
Known liver disease	8 (4.1)
Old DVT	3 (1.5)
Peptic ulcer	5 (2.6)
Autoimmune disease	3 (1.5)
*Clinical presentation*	
HF functional class	
NYHA class III	15 (7.7)
NYHA class IV	163 (83.6)
Cardiogenic shock	17 (8.7)
Chest pain	117 (60.3)
SBP (mmHg)	118.2 ± 28
DBP (mmHg)	72.9 ± 15.7
HR (bpm)	95.7 ± 23.4

BMI: body mass index; HF: heart failure; HTN: hypertension; DM: diabetes mellitus; CAD: coronary artery disease; PCI: percutaneous coronary intervention; CABG: coronary artery bypass graft; ESKD: end-stage kidney disease; CVA: cerebrovascular accident; PAD: peripheral arterial disease; DVT: deep vein thrombosis; DKA: diabetic ketoacidosis; NYHA: New York Heart Association; SBP: systolic blood pressure; DBP: diastolic blood pressure; HR: heart rate

With a mean LVEF of 31% ± 8.7, HFrEF and HFmrEF were found in 76.9% and 23.1% of the patients, respectively. CAD (80.5%) and idiopathic CMP (14.4%) were the two most commom etiologies. Other less frequent etiologies included thyrotoxicosis, PPCM, and VHD. More than 50% of the patients presented with ADHF, 41% had new-onset HF, and 8.2% were in cardiogenic shock. ACS, which was the primary presentation in 43.9% of the patients, chest infection (13.8%) and arrhythmias (7.7%) were the most frequent triggering factors for the AHF.

## Primary and secondary cardiovascular outcomes in the final study population

The in-hospital mortality rate during the index hospitalization was 4% among the 174 patients who were followed up. The primary outcome, which was a composite of CV mortality or HHF, occurred in 42% of patients with a mean time from enrolment of 4.2 ± 3.3 months. CV death and HHF occurred in 30.5% and 23.9% of the patients, respectively. ASCV events occurred in 7.4% of the cases. The median CCU stay was 12 (IQR = 13.3) days, while the median LOS was 6 (IQR = 9) days. (Table 2).

**Table 2:** Primary and secondary cardiovascular outcomes in the final study population

Variables	Number (%) or mean ± SD (n = 174)
*Primary endpoint*	
HHF or CV Death	73 (42)
Time interval from enrollment to HHF or death (months)	4.2 ± 3.3
*Secondary endpoints*	
Heart failure hospitalization	34 (23.9)
CV death	53 (30.5)
ASCV event	13 (7.4)
ACS	7 (4)
CVA	3 (1.7)
Vascular	3 (1.7)
Hospitalization for other causes	8 (4.6)
LOS (days)	Median 6 (IQR = 9)
CCU (days)	Median 12 (IQR = 13.3)
Cardiac arrest during index admission	8 (4.6)
Death during index admission	7 (4)
*Mode of death*	
Mechanical	30 (57.7)
SCD	21 (40.4)
Other	1 (1.9)
Time interval from enrollment to first ASCV event (months)	7.5 ± 4.4

HHF: hospitalization for heart failure; CV: cardiovascular; SCD: sudden cardiac death; ASCV event: atherosclerotic cardiovascular event; ACS: acute coronary syndrome; CVA: cerebrovascular accident;

## Clinical Characteristics of patients with right ventricular dysfunction

Although patients with RVD were younger, they had a significantly longer duration of HF. A history of previous revascularization or chemotherapy and a higher heart rate were more common in the RVD group, while chest pain, HTN, and CAD were more prevalent in those with normal RV function. Other comorbidities were similar between the two groups. RVD patients had significantly lower hemoglobin levels, total leukocyte count, and a better lipid profile, except for high-density lipoprotein-cholesterol (HDL-C). As for ECG findings, AF/flutter was significantly associated with RVD, whereas changes in the ST segment and T wave were more significant in the normal RV group. (Table 3).

**Table 3:** Clinical profile of patients with right ventricular dysfunction versus those with normal right ventricular function

Variables	No RVD Number (%) or mean ± SD (n = 88)	RVD Number (%) or mean ± SD (n = 86)	*P* value
Age	59.6 ± 10	55.9 ± 11.9	**0.03**
BMI (kg/m^2^)	28.8 ± 15.7	27.7 ± 5.2	0.8
Male gender	66 (75)	65 (75.6)	0.093
*Smoking status*			
Non-smoker	29 (33)	30 (34.9)	0.059
Current smoker	45 (51.1)	31 (36)	
Ex-smoker	14 (15.9)	25 (29.1)	
*HF duration*			
< 1 month	45 (64.3)	20 (28.2)	** < 0.001**
> 1 month—< 1 year	13 (18.6)	24 (33.8)	
1–5 year(s)	9 (12.9)	20 (28.2)	
> 5 years	3 (4.3)	7 (9.9)	
*Comorbidities*			
HTN	52 (59.1)	38 (44.2)	**0.049**
DM	47 (53.4)	48 (55.8)	0.75
CAD	81 (94.2)	63 (75.9)	**0.001**
CAD duration < 1 month	44 (50)	9 (10.5)	** < 0.001**
Previous PCI/CABG	17 (19.3)	28 (32.6)	**0.046**
Previous cardiac arrest	2 (2.3)	1 (1.2)	0.57
Chronic chest disease	19 (21.6)	20 (23.3)	0.79
Renal impairment	38 (43.2)	36 (41.9)	0.86
ESKD on regular dialysis	2 (2.3)	2 (2.3)	0.98
Previous CVA	8 (9.1)	14 (16.3)	0.15
Previous exposure to chemotherapy	0	4 (4.7)	**0.04**
Infection	23 (26.1)	23 (26.7)	0.93
Thyroid disorders	3 (3.4)	3 (3.5)	0.98
PAD	7 (8)	9 (10.5)	0.57
Known liver disease	2 (2.3)	6 (7)	0.14
Old DVT	1 (1.1)	1 (1.2)	0.99
Peptic ulcer	2 (2.3)	2 (2.3)	0.98
DKA	2 (2.3)	0	0.16
Autoimmune disease	0	3 (3.5)	0.12
*Clinical presentation*			
Chest pain	72 (81.8)	36 (42.4)	** < 0.001**
*HF functional class*			
NYHA class III	7 (8)	7 (8.1)	
NYHA class IV	71 (80.7)	73 (84.9)	**0.066**
Cardiogenic shock	10 (11.4)	6 (7)	
SBP (mmHg)	121.1 ± 31.7	114.5 ± 24	0.14
DBP (mmHg)	74 ± 17.5	71.2 ± 14.6	0.26
HR (bpm)	91.5 ± 20.2	99.7 ± 25.9	**0.02**
*Laboratory*			
RBS (mg/dL)	196.9 ± 111	180 .9 ± 82.3	0.4
Creatinine (mg/dL)	1.5 ± 1.0	1.6 ± 1.3	0.55
Na (mmol/L)	132.6 ± 20.6	133.8 ± 6.1	0.61
K (mmol/L)	4.5 ± 0.8	4.3 ± 0.7	0.1
Hb (g/dL)	13.1 ± 2.3	12 ± 1.9	**0.001**
TLC (× 10^3^/L)	11.2 ± 4.3	8.5 ± 3.1	** < 0.001**
Platelets (× 10^3^/L)	278 ± 81.6	256.3 ± 115.1	0.16
TC (mg/dL)	169 ± 52.5	128.2 ± 43	** < 0.001**
TG (mg/dL)	146.7 ± 109.9	95.7 ± 45.2	**0.001**
HDL-C (mg/dL)	37.9 ± 15	30.1 ± 8.5	**0.001**
LDL-C (mg/dL)	103.8 ± 44.7	78.3 ± 34.9	** < 0.001**
*ECG*			
Rhythm			
Sinus	85 (96.6)	65 (75.6)	** < 0.001**
AF/A. FL	3 (3.4)	20 (24.4)	
Conduction abnormalities			
None	68 (81)	59 (72.8)	
LBBB	10 (11.9)	15 (18.5)	0.44
RBBB	6 (7.1)	7 (8.6)	

Significant *P* values are given in bold (*P* value < 0.05)

RVD: Right ventricular dysfunction; BMI: body mass index; HF: heart failure; HTN: hypertension; DM: diabetes mellitus; CAD: coronary artery disease; PCI: percutaneous coronary intervention; CABG: coronary artery bypass graft; ESKD: end-stage kidney disease; CVA: cerebrovascular accident; PAD: peripheral arterial disease; DVT: deep vein thrombosis; DKA: diabetic ketoacidosis; NYHA: New York Heart Association; SBP: systolic blood pressure; DBP: diastolic blood pressure; HR: heart rate; RBS: random blood sugar; Na: sodium; K: potassium; Hb: hemoglobin; TLC: total leucocytic count; TC: total cholesterol; TG: triglycerides; HDL-C: high density-lipoprotein-cholesterol; LDL-C: low density-lipoprotein-cholesterol; ECG: electrocardiography; A. FL: atrial flutter; A.F.: atrial fibrillation; BBB: bundle branch block; LBBB: left bundle branch block; RBBB: right bundle branch block

## Echocardiographic features of right ventricular dysfunction (Fig. 3)

**Fig. 3:**
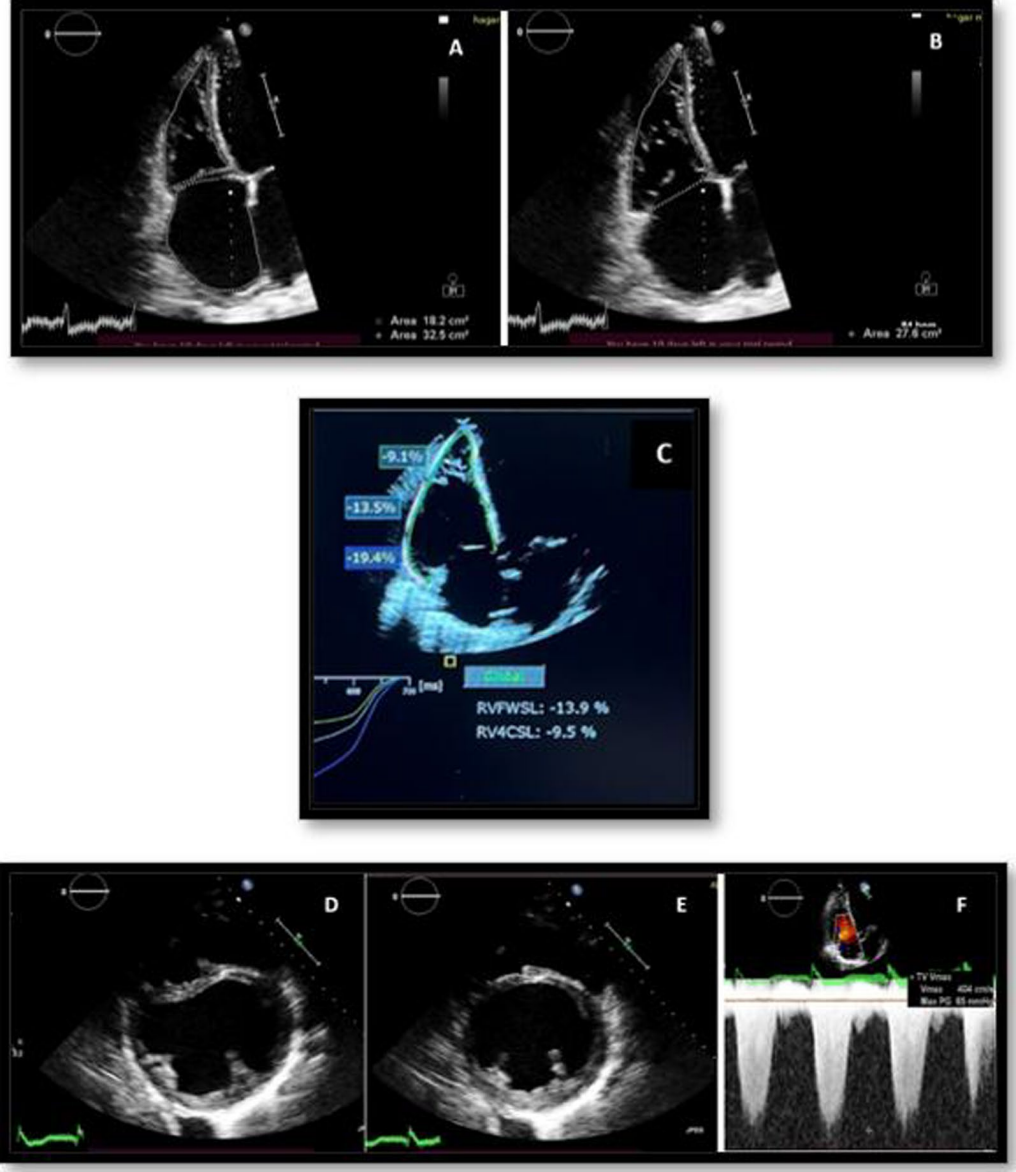
Echocardiographic findings in a patient with ischemic cardiomyopathy. **A** & **B** Dilated right ventricular end-systolic area (ESA) and end-diastolic area (EDA), as well as dilated right atrium (RA); & impaired RV fractional area change (FAC = 34%); **C** Impaired RV longitudinal strain; longitudinal RV free wall strain (RV FWS = − 13.9) and right ventricular four-chamber strain (RV4CSL = − 9.5%); **D** & **E** Parasternal short axis view, at papillary muscle level, showing septal shift towards the left ventricle, mainly during systole; **F** Continuous wave Doppler of the tricuspid valve showing high regurgitant jet velocity and estimated PASP = 65 mmHg

## Left ventricle

RVD patients had significantly higher linear and volumetric LV dimensions. They also had lower LVEF. The regional wall motion abnormalities (RWMA) were similar in both groups. However, the RVD group had significantly more interventricular septal hypokinesia and dyskinesia, while the normal RV function group had more interventricular septal akinesia. (Table 4).

**Table 4:** Comparison of patients with right ventricular dysfunction and those with normal right ventricular function regarding echocardiographic findings

Variables	No RVD Number (%) or mean ± SD (n = 88)	RVD Number (%) or mean ± SD (n = 86)	*P* value
*Left heart size*			
LVEDD (mm)	57.3 ± 9.9	62.3 ± 9.6	**0.001**
LVESD (mm)	45.4 ± 9.9	52.2 ± 9.4	** < 0.001**
SWT (mm)	9.8 ± 1.9	9.0 ± 2.1	**0.004**
PWT (mm)	9.7 ± 1.6	9.1 ± 1.9	**0.04**
LV EDV (ml)	151.4 ± 65.3	189 ± 55.4	** < 0.001**
LV ESV (ml)	101.7 ± 53.6	138.1 ± 48.3	** < 0.001**
LA ESV (ml)	58 ± 27.2	80.3 ± 28.4	** < 0.001**
*LV function*			
EF % (Simpson)	34.9 ± 8.1	27.8 ± 7.9	** < 0.001**
E/A	1.27 ± 0.7	2.2 ± 0.9	** < 0.001**
RWMA IVS	84 (96.6)	86 (100)	0.25
Hypokinetic	23 (26.1)	31 (36)	**0.003**
Akinetic	58 (65.9)	42 (58.8)	
Dyskinetic	3 (3.4)	13 (15.1)	
RWMA Overall	88 (100)	86 (100)	1.0
Hypokinetic	22 (25)	28 (32.6)	**0.017**
Akinetic	62 (70.5)	45 (52.3)	
Dyskinetic	4 (4.5)	13 (15.1)	
*Right heart size*			
RVWT (PLAX) (mm)	4.2 ± 0.7	4.4 ± 0.8	0.12
RVWT (subcostal) (mm)	4.6 ± 0.8	4.8 ± 1.0	0.11
RVOT (PLAX) (mm)	31.5 ± 4.9	37.4 ± 5.9	** < 0.001**
RVOT 1 (mm)	31.2 ± 4.7	36.9 ± 5.6	** < 0.001**
RVOT 2 (mm)	25.9 ± 3.6	28.7 ± 4.6	** < 0.001**
Main PA (mm)	22.1 ± 3.6	24 ± 4.1	**0.003**
RVD 1 (mm)	36.6 ± 6.8	46.5 ± 9	** < 0.001**
RVD 2 (mm)	24.9 ± 6.0	34 ± 8.1	** < 0.001**
RVD 3 (mm)	71.2 ± 11.2	79.4 ± 8.3	** < 0.001**
RV EDA (cm^2^)	18.7 ± 7.1	27.6 ± 7.3	** < 0.001**
RV ESA (cm^2^)	10.3 ± 5.2	20.6 ± 6.4	** < 0.001**
RA width (mm)	35.1 ± 6.5	44.4 ± 9.8	** < 0.001**
RA length (mm)	43.5 ± 8.3	51.3 ± 10.1	** < 0.001**
RA area (cm^2^)	14.1 ± 4.9	21.2 ± 7.4	** < 0.001**
RVH	7 (8)	10 (11.6)	0.42
Dilated RVOT	38 (44.7)	75 (87.2)	** < 0.001**
Dilated RV	13 (14.8)	57 (66.3)	** < 0.001**
Dilated RVOT & RV	42 (47.7)	75 (87.2)	** < 0.001**
*RV function*			
TAPSE (mm)	20.7 ± 5.8	12.7 ± 3.6	** < 0.001**
RV S' (cm/s)	12.4 ± 2.3	8.2 ± 2.3	** < 0.001**
RV FAC	46.3 ± 8.9	25.2 ± 7.5	** < 0.001**
RVD	0	86 (100)	** < 0.001**
RVD & Dilated RV	0	57 (66.3)	** < 0.001**
RVD & Dilated RVOT & RV	0	72 (83.7)	** < 0.001**
RIMP	0.47 ± 0.05	0.72 ± 0.19	** < 0.001**
RV FWS	− 21.3 ± 7.8	− 13.2 ± 5.8	** < 0.001**
RV GLS	− 15.7 ± 6.9	− 10.8 ± 5.2	** < 0.001**
*Estimated right-sided pressures*			
RVSP (mmHg)	34.4 ± 13.1	39.9 ± 13.1	**0.03**
EPASP (mmHg)	42.4 ± 14.7	51.4 ± 14.3	**0.001**
RAP (mmHg)	6.4 ± 4.7	10.8 ± 5.3	** < 0.001**
IVC_EXP_ (mm)	17.4 ± 6.6	22.1 ± 6.9	** < 0.001**
IVC_INS_ (mm)	7.1 ± 9.2	15.2 ± 10.8	** < 0.001**
*Valvular abnormalities*			
Tricuspid regurgitation			
None	21 (24.7)	6 (7)	** < 0.001**
Mild	45 (53)	32 (37.2)	
Moderate	13 (15.3)	22 (25.6)	
Severe	6 (7.1)	26 (30.2)	
Mitral regurgitation			
None	14 (16.1)	4 (4.7)	** < 0.001**
Mild	44 (50.5)	24 (27.9)	
Moderate	20 (20.3)	23 (26.7)	
Severe	9 (10.3)	35 (40.7)	
Mitral Stenosis			
None	87 (98.9)	84 (97.7)	0.6
Mild	0	0	
Moderate	0	1 (1.2)	
Severe	1 (1.1)	1 (1.2)	
Aortic regurgitation			
None	72 (81.8)	69 (80.2)	0.75
Mild	16 (18.1)	16 (18.6)	
Moderate	0	1 (1.2)	
Severe	0	0	
Aortic stenosis			
None	86 (97.7)	86 (100)	0.37
Mild	2 (2.2)	0	
Moderate	0	0	
Severe	1 (1.1)	0	
Pericardial effusion			
None	80 (90.9)	75 (87.2)	0.51
Mild	8 (9.1)	10 (11.6)	
Moderate	0 (0)	1 (1.2)	
Severe	0	0	
Intracardiac thrombus	11 (12.5)	5 (5.8)	0.13

Significant *P* values are given in bold (*P* value < 0.05)

LV: left ventricle; LVEDD: LV end-diastolic diameter; LVESD: LV end-systolic diameter; SWT: septal wall thickness; PWT: posterior wall thickness; EDV: end-diastolic volume; ESV: end-systolic volume; Ao: aorta; LA: left atrium; EF: ejection fraction; RWMA: regional wall motion abnormalities; IVS: interventricular septum; PLAX: parasternal long axis; RVOT: right ventricular outflow tract; RVOT 1: proximal RVOT diameter in the parasternal short axis view; RVOT 2: distal RVOT diameter in the parasternal short axis view; PA: pulmonary artery; RV: right ventricle; RVD 1: RV basal diameter; RVD 2: RV mid diameter; RVD 3: RV length; RVWT: RV wall thickness; EDA: end-diastolic area; ESA: end-systolic area; RA: right atrium; RVH: RV hypertrophy; TAPSE: tricuspid annular plane systolic excursion; S': tissue Doppler derived systolic excursion velocity; FAC: fractional area change; RVD: right ventricular dysfunction; RIMP: right ventricular index of myocardial performance; FWL: free wall longitudinal strain; GLS: global longitudinal strain; RVSP: RV systolic pressure; EPASP: estimated pulmonary artery systolic pressure; RAP: right atrial pressure; IVCEXP: inferior vena cava diameter during expiration; IVCINS: inferior vena cava diameter during inspiration

## Right ventricle

Excluding the RV wall thickness, all right-sided dimensions were larger and right-sided pressures were significantly higher in the RVD group. Although the mean RV GLS was reduced in both groups, it was significantly lower in the RVD group. (Table 4).

## Valve affection

More severe grades (moderate and severe) of mitral and tricuspid regurgitation were significantly associated with RVD. By contrast, absent mitral and/or tricuspid regurgitation or mild grades were more common in the normal RV group. However, both groups had similar aortic valve dysfunction and stenotic lesions of the mitral and tricuspid valves. (Table 4).

## Angiographic data

The RVD group had statistically more coronary artery bypass graft surgeries (14% vs. 4.5%) and less diagnostic coronary angiography and percutaneous interventions (42.7% vs. 65.5%) than the normal RV group (*p* = 0.03). Normal epicardial coronary anatomy and multivessel disease were more prevalent in the RVD group (19% vs. 3.1%, and 40.5% vs. 34.4%, respectively). In contrast, single and 2-vessel diseases were significantly more common in the normal RV group (29.7% vs. 14.3% and 32.8% vs. 26.2%, respectively; *p* = 0.02).

## Medical treatment

Diuretic therapy, spironolactone, and digoxin were more commonly prescribed in the RVD group, whereas statins, antiplatelet drugs, and angiotensin-converting enzyme inhibitors were more common in the normal RV function group. However, total cholesterol, triglyceride, and low-density lipoprotein cholesterol levels were also significantly higher in the latter group. (Table 5).

**Table 5:** Comparison of patients with right ventricular dysfunction and those with normal right ventricular function regarding medical treatment

Medical treatment	No RVD Number (%) or mean ± SD (n = 88)	RVD number (%) or mean ± SD (n = 86)	**P** value
ASA	78 (89.7)	48 (58.5)	< 0.001
Clopidogrel	57 (65.5)	25 (30.5)	< 0.001
Ticagrelor	8 (9.2)	1 (1.2)	0.021
Beta-blocker	67 (77.9)	55 (67.9)	0.15
ACEI	57 (67.1)	39 (48.1)	0.014
ARB	3 (3.5)	2 (2.5)	0.69
Spironolactone	31 (36.5)	57 (70.5)	< 0.001
Sacubitril/valsartan	4 (4.7)	7 (8.5)	0.32
Diuretic	51 (59.3)	74 (90.2)	< 0.001
Loop diuretic alone	49 (96.1)	69 (93.2)	0.5
Combination	2 (3.9)	5 (6.8)	
CCB	6 (7.1)	2 (2.4)	0.16
Ivabradine	15 (17.6)	8 (9.8)	0.14
Nitrates	11 (12.9)	7 (8.5)	0.36
Digoxin	2 (2.4)	17 (20.7)	< 0.001
Amiodarone	7 (8.2)	6 (7.1)	0.79
Proton pump inhibitor	80 (93)	80 (95.2)	0.54
Insulin	16 (19)	25 (30.5)	0.09
Anticoagulant	24 (27.3)	25 (29.4)	0.76
Statins	79 (90.8)	62 (75.6)	0.008
Ezetimibe	6 (7)	3 (3.6)	0.33

ASA: acetylsalicylic acid (aspirin); ACEI: angiotensin-converting enzyme inhibitor; ARB: angiotensin receptor blocker; CCB: calcium channel blocker

## Characteristics of heart failure in right ventricular dysfunction patients

Idiopathic dilated cardiomyopathy (DCM) was the most common etiology in the RVD group, whereas CAD was the most common etiology in the normal RV group (*p* = 0.005). HFrEF was significantly associated with RVD (89.5% vs. 65.9%), whereas HFmrEF was more prevalent in the normal RV function group (34.1% vs. 10.5%); (*p* < 0.001).

Acute decompensated HF (ADHF) was the most common presentation of AHF in patients with RVD, which correlated with the longer duration of HF in this group. In contrast, de novo HF and cardiogenic shock were significantly more prevalent in the normal RV group, that also had a significantly higher incidence of ACS. (Table 6).

**Table 6:** Comparison of patients with right ventricular dysfunction and those with normal right ventricular function regarding characteristics of heart failure

HF classification	No RVD Number (%) or mean ± SD (n = 88)	RVD number (%) or mean ± SD (n = 86)	*P* value
*Etiology*			
CAD only	82 (93.2)	60 (69.8)	0.005
Idiopathic	4 (4.5)	17 (19.8)	
Peripartum	0	1 (1.2)	
Endocrine	1 (1.1)	1 (1.2)	
Infective	0	4 (4.7)	
VHD only	0	2 (2.3)	
CAD + VHD	1 (1.1)	1 (1)	
*Clinical phenotypes*			
ADHF	33 (37.5)	65 (75.6)	< 0.001
De-novo AHF	46 (52.3)	15 (17.4)	
Cardiogenic shock	9 (10.2)	6 (7)	
*Precipitating factors*			
ACS	62 (70.5)	17 (19.8)	< 0.001
Chest infection	9 (10.2)	12 (14)	0.45
Arrhythmia	5 (5.7)	7 (8.1)	0.52
AKI	5 (5.7)	8 (9.3)	0.36
Non-adherence to treatment	0	2 (2.3)	0.15

CAD: coronary artery disease; VHD: valvular heart disease; ADHF: acute decompensated heart failure; AHF: acute heart failure; LVEF: left ventricular ejection fraction; ACS: acute coronary syndrome; AKI: acute kidney injury

## Primary and secondary cardiovascular outcomes

The primary endpoint and its components were significantly more common in the RVD group. The length of CCU stay was also significantly longer in the RVD group (43 vs. 32 above the median, p = 0.02), whereas the median LOS was similar in the two groups (41 vs. 34 above the median, *p* = 0.15). Additionally, no significant differences were found between the 2 groups in the incidence of ASCV events, PE, or arrhythmias during the follow-up period or in in-hospital death, total LOS, or hospitalization for other causes. (Table 7).

**Table 7:** Primary and secondary outcomes in patients with right ventricular dysfunction versus those with normal right ventricular function

Variables	No RVD Number (%) or mean ± SD (n = 88)	RVD Number (%) or mean ± SD (n = 86)	*P* value
*Primary endpoint*			
HHF or CV Death	28 (31.8)	45 (52.3)	**0.006**
Time interval from enrollment to HHF or death (months)	3.8 ± 3.5	4.5 ± 3.2	0.4
*Secondary endpoints*			
CV death	20 (22.7)	33 (38.4)	**0.025**
HHF	12 (16.2)	22 (32.4)	**0.047**
All-cause death	3 (3.4)	4 (4.7)	0.68
Hospitalization for any cause	3 (3.4)	5 (5.8)	0.49
ASCV event	7 (4)	6 (3.4)	0.69
ACS	3 (3.4)	4 (4.7)	
CVA	2 (4)	1 (16.7)	0.5
Vascular	2 (28.6)	1 (16.7)	
Arrhythmias	8 (9.1)	8 (9.3)	0.96
Time interval from enrollment to first ASCV event (months)	7.5 ± 5.8	7.4 ± 2.6	0.97

Significant *P* values are given in bold (*P* value < 0.05)

HHF: hospitalization for heart failure; CV: cardiovascular; SCD: sudden cardiac death; ASCV event: atherosclerotic cardiovascular event; ACS: acute coronary syndrome; CVA: cerebrovascular accident; LOS: length of hospital stay; CCU: cardiac intensive care unit

## Multivariate regression analysis

**Fig. 4:**
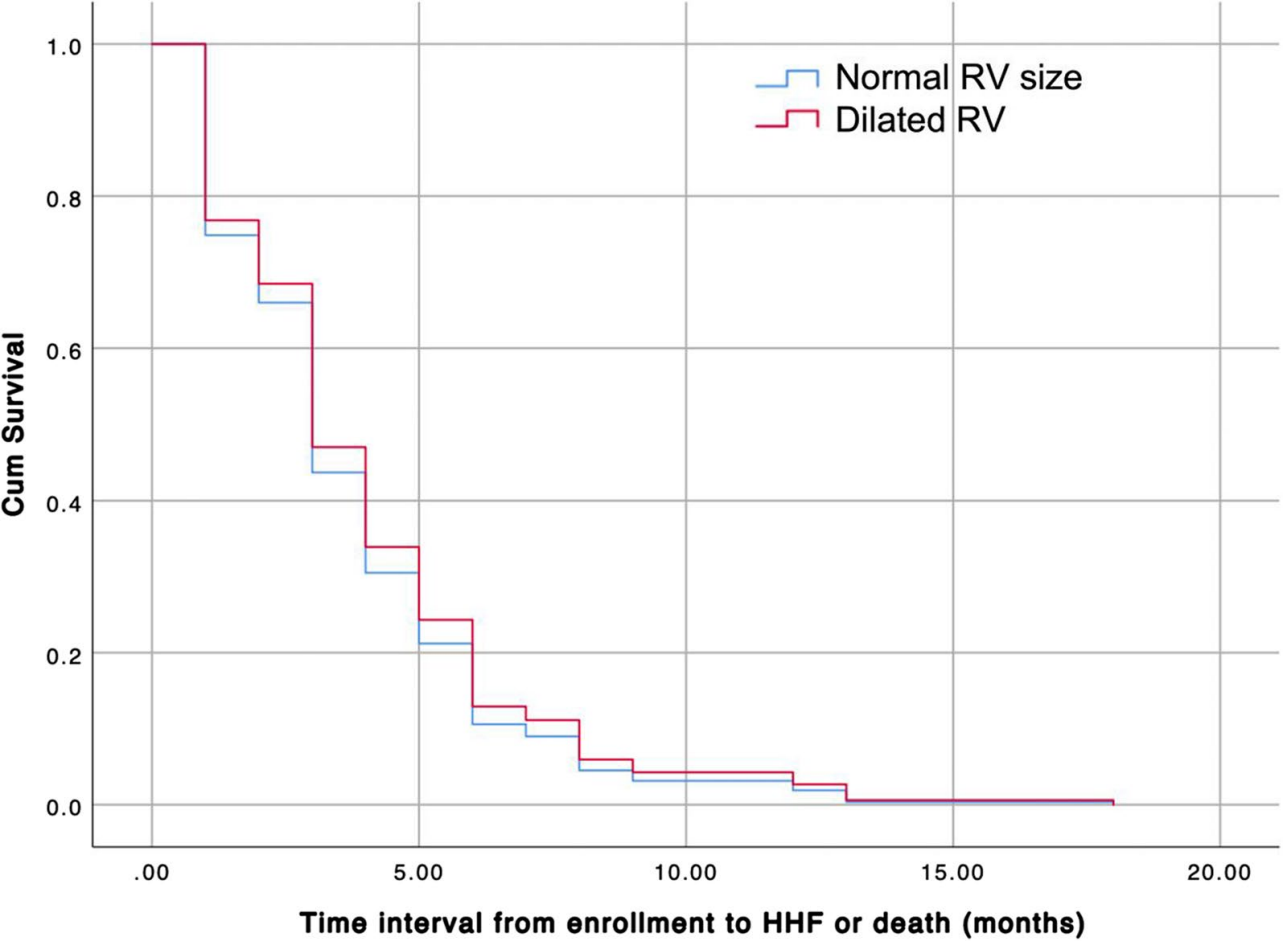
Cox Regression Curve of Normal and Dilated RV in predicting the primary endpoint

In multivariate Cox regression analysis, the only significant predictor of the primary outcome was the RV GLS. (Table 8 and Fig. 4).

**Table 8:** Cox regression analysis of predictors of the primary outcome

Variable	HR	95% CI	*P* value
RV GLS	1.07	1.01–1.14	**0.014**
Age	1.0	0.97–1.03	0.97
RV S'	1.02	0.91–1.14	0.77
TAPSE	0.995	0.95–1.04	0.8
CAD history	1.16	0.5–2.7	0.73
RV FAC	1.02	0.98–1.07	0.35
RV dilation	0.9	0.32–2.61	0.86

Significant *P* value is given in bold (*P* value < 0.05)

RV: right ventricular; GLS: right ventricular global longitudinal strain; S': tissue Doppler derived systolic excursion velocity; TAPSE: tricuspid annular plane systolic excursion; CAD: coronary artery diseases; FAC: fractional area change

## Discussion

In this study, RVD was observed in almost half of the patients with AHF. It predicted CV death and HHF and was associated with prolonged hospital stays.

Few studies have addressed the prognostic value of RVD in AHF patients. Furthermore, comparisons between these studies are confounded by the lack of consistency in the methodology used to diagnose RVD, different inclusion criteria of the study cohorts, and variable follow-up periods.

## Characteristics and presentations of AHF patients

The mean age of our patients was 57.7 ± 10.9 years; most were males (74.9%). HTN was present in 49.7% of patients, DM in 54.4%, and chronic chest disease in 23.6%. The most common etiology of HF was CAD, followed by DCM (80.5% and 13.3% of the cases, respectively). The clinical presentation was ADHF in 60% of the cases and cardiogenic shock in 8.2%. The most common precipitating factors for AHF were ACS (43.9%), chest infection (13.8%), and arrhythmias (7.7%), and most patients were males. A similar clinical profile was seen in the ESC-HF-LT Registry [21], which comprised 6629 AHF patients from 21 European and Mediterranean countries: females represented 37.4% of cases, and the most common clinical presentation was ADHF (61.1%). CAD was also the most common etiology of HF, and DCM was present in 13.6% of the patients. However, the patients were older (mean age = 69.35 ± 12.98). ACS was less prevalent (14.4% vs. 43.9%, respectively), and the most common precipitating factors for HF were arrhythmias, especially AF (43%), followed by myocardial ischemia (30%) and infections (20%). Cardiogenic shock was also much less common (2.9 vs. 8.2%), probably because there were fewer patients with HFrEF (25.2% vs. 76.9%) [21].

## Right ventricular dysfunction in acute heart failure

We diagnosed RVD in 48.7% of the patients with AHF and LVEF < 50%. A previous study on 84 patients reported a similarly high prevalence of RVD (46%) in AHF patients using a TAPSE cut-off of < 17 mm. [22] We defined RVD as TAPSE < 17 mm, RV S' < 9.5 cm/s and/or RV FAC < 35%, and it was significantly associated with a worse primary composite outcome (CV death and HHF). Similarly, Ashcroft et al. [23] study on 418 AHF patients showed that the parameters of RV function, including TAPSE and RV FAC, were predictors of poor outcomes, namely all-cause mortality, which was the primary endpoint after a two-year-follow-up period. Awad et al. [24] demonstrated that RV function assessment by FAC, TAPSE, or RV S' also predicted poor outcomes. However, they included a different cohort of patients presenting with acute myocardial infarction (AMI) only. The primary endpoints were major adverse cardiac events: death, hospitalization for recurrent ischemia, reinfarction, HHF, arrhythmias needing hospitalization, and ischemic stroke. The follow-up period was only 30 days, and the mean LVEF was higher than in our study [24].

## Right ventricular dysfunction as a predictor of outcome

In our study, RV function assessment by strain was a better predictor of the primary outcome than the other routinely used parameters; thus, subtle changes in RV function could still predict a worse effect on the outcome. Multivariate analysis revealed that only RV GLS continued to significantly predict poor outcomes in patients with AHF and LVEF < 50%. In contrast, another study showed that RV GLS predicted lower event-free survival in patients with normal LV, but not in those with LV systolic dysfunction. However, this study was conducted on 282 patients with inferior STEMI; only 30% had impaired LVEF [25]. On the other hand, in accordance with our results, Carluccio et al. [26] studied 200 patients with HFrEF for about 28 months and demonstrated that RV strain predicted death and HHF better than FAC, RV S', and TAPSE –the latter was preserved in all patients. However, in this study, the RV FWS was an independent predictor of outcomes, whereas in our study, it was the RV GLS. In another study, RV FAC, TAPSE, and RV strain were able to predict the composite endpoint of all-cause death, reinfarction, and HHF in 621 AMI patients, after a mean follow-up period of 24 months. In multivariate analysis, only RV FWS and RV FAC remained independent predictors of the composite endpoint. Nevertheless, the predictive value of RV strain at a cutoff value of < -2.1% was even more potent than that of FAC. So, like our study, they showed the incremental value of speckle tracking echocardiography over conventional RV functional parameters [27].

In our study, RVD also correlated with lower LVEF, which is similar to the findings of another study on new-onset HF, which showed that HFrEF had more RVD than HFmrEF and heart failure with preserved ejection fraction (HFpEF) (43.5% vs. 30.7%) [28]. In agreement with our findings, a CMR study conducted on patients with DCM and a mean LVEF of 32.9% ± 11.6 showed that reduced LVEF was one of the independent predictors of RVD, which in turn was a strong predictor of cardiac mortality [29]. Interestingly, Movahed et al., using gated equilibrium radionuclide angiography, showed an increasing correlation between RVEF and LVEF with decreasing LVEF and RVEF –the strongest correlation being in those with LVEF and RVEF < 30%, while there was no correlation in patients with normal biventricular EF [30].

In our study, the prevalence of CAD and dyslipidemia was higher in patients with normal RV function. This may be explained by the higher proportion of patients with ACS and de novo AHF with uncontrolled atherosclerotic risk factors. In contrast, RVD was more commonly associated with idiopathic DCM, longer HF duration, and ADHF. In contrast to these findings, Parcharidou et al. [31] reported that RVD was more noticeable in patients having ICM than in those with DCM. Although the mean LVEF was comparable to that in our study (29.3 ± 8 in the DCM group and 27.8 ± 6.3 in the ICM group), they excluded patients with AF, recent myocardial infarction, unstable angina, or severe HTN. On the other side, in accordance with our study, D'Andrea et al. [32] reported that RVD, defined by impaired RV GLS and assessed in 110 patients who were candidates for cardiac resynchronization therapy device implantation, was more pronounced in idiopathic than in ICM patients. Similarly, Grebe et al. [33] demonstrated that RV function, assessed using CMR in 141 patients with LVEF < 35%, was significantly worse in patients with DCM than in those with ICM.

The primary outcome, in our study, occurred in 42% of our patients after a follow-up period of 4.2 ± 3.3 months; CV death occurred in 30.5% and HHF in 23.9% of the patients. These rates are much higher than those seen in the ESC-HF-LT Registry [21]: 23.6% all-cause mortality (51.7% due to CV deaths), 18.7% HHF, and 36% combined endpoint, but their follow-up period was longer (one year). Nevertheless, the in-hospital death rate in our study was lower (4% vs. 4.9%) –both numbers are comparable to the rates reported globally (4–10%). In contrast, in the OPTIMIZE-HF registry, rehospitalization alone and the composite endpoint of death or rehospitalization occurred in 29.6% and 36% respectively, during the 60 to 90 days after hospital discharge. However, their in-hospital death rate was 3.8%, which is lower than ours [34].

As reported in literature, atrial tachyarrhythmia is the most common arrhythmia observed in patients with RVF. In our study, RVD was significantly associated with AF, larger LA dimensions, and increased end-systolic volumes. Similarly, Aziz et al. [35] showed that RVD was a strong predictor of AF in 904 patients with ADHF. RVD was also a predictor of a twofold higher composite endpoint of HHF and all-cause death when compared with those with normal RV [35].

Finally, we found that RVD was significantly associated with longer CCU stays but not with total hospital stays. In contrast, Yamin et al. [36] reported that RV function assessed using TAPSE was a significant predictor of hospital LOS. Although, similarly to our study, they had a high prevalence of CAD and LVEF < 40% (76.5% and 74.1%, respectively), they excluded patients with evidence of ACS, severe TR, and those who died during admission [36].

Our study had some limitations. It was performed at a single center on a relatively small number of patients. Most participants were male and had CAD, which may limit the value of our findings when extrapolated to female and non-ischemic patients. Finally, we did not compare our results to those of 3DE or CMR, which are better modalities for evaluating RV function. However, most of our patients were critically ill, and it would have been difficult to perform a lengthy procedure or one that required patient transfer to the radiology unit.

## Conclusions

RVD, assessed using conventional echocardiographic methods (TAPSE, FAC, and RV S') and strain analysis, was a predictor of the composite of CV death and HHF in patients admitted with left-sided AHF. In multivariate analysis, only RV GLS remained an independent predictor of outcomes. RVD was also significantly associated with a longer CCU stay during the index admission.

## Data Availability

The data set supporting the results and conclusions of this article will be available from the corresponding author on request.

## Abbreviations

ACS: Acute coronary syndrome
ADHF: Acute decompensated heart failure
AMI: Acute myocardial infarction
BP: Blood pressure
CAD: Coronary artery disease
CCU: Cardiac care unit
CMR: Cardiac magnetic resonance
CV: Cardiovascular
CVA: Cerebrovascular accident
DCM: Dilated cardiomyopathy
DM: Diabetes mellitus
EF: Ejection fraction
FAC: Fractional area change
FWS: Free wall strain
GLS: Global longitudinal strain
HHF: Hospitalization for heart failure
HF: Heart failure
HFmrEF: Heart Failure with mildly reduced ejection fraction
HFpEF: Heart failure with preserved ejection fraction
HFrEF: Heart failure with reduced ejection fraction
HTN: Hypertension
ICM: Ischemic cardiomyopathy
LA: Left atrial/atrium
LOS: Length of stay
LV: Left ventricular/ventricle
LVEF: Left ventricular ejection fraction
NT pro-BNP: N-terminal pro-brain natriuretic peptide
RA: Right atrial/atrium
RV: Right ventricular/ventricle
RVOT: Right ventricular outflow tract
RVD: Right ventricular dysfunction
RVEF: Right ventricular ejection fraction
RWMA: Regional wall motion abnormalities
STEMI: ST elevation myocardial infarction
TAPSE: Tricuspid annular plane systolic excursion
TDI: Tissue Doppler imaging
2DE: Two-dimensional transthoracic echocardiography
3DE: Three-dimensional echocardiography
